# Quantitative Fluorescence Imaging of Perfusion—An Algorithm to Predict Anastomotic Leakage

**DOI:** 10.3390/life12020249

**Published:** 2022-02-08

**Authors:** Sanne M. Jansen, Daniel M. de Bruin, Leah S. Wilk, Mark I. van Berge Henegouwen, Simon D. Strackee, Suzanne S. Gisbertz, Ed T. van Bavel, Ton G. van Leeuwen

**Affiliations:** 1 Amsterdam UMC, Unit L0, Department of Biomedical Engineering & Physics, Faculty of Medicine, University of Amsterdam, Meibergdreef 9, 1105 AZ Amsterdam, The Netherlands; d.m.debruin@amsterdamumc.nl (D.M.d.B.); l.s.wilk@amsterdamumc.nl (L.S.W.); e.t.vanbavel@amsterdamumc.nl (E.T.v.B.); t.g.vanleeuwen@amsterdamumc.nl (T.G.v.L.); 2 Amsterdam UMC, Unit G4, Department of Plastic, Reconstructive and Hand Surgery, Faculty of Medicine, University of Amsterdam, Meibergdreef 9, 1105 AZ Amsterdam, The Netherlands; s.d.strackee@amsterdamumc.nl; 3 Amsterdam UMC, Department of Surgery, Cancer Center Amsterdam, University of Amsterdam, Meibergdreef 9, 1105 AZ Amsterdam, The Netherlands; m.i.vanbergehenegouwen@amsterdamumc.nl (M.I.v.B.H.); s.s.gisbertz@amsterdamumc.nl (S.S.G.)

**Keywords:** perfusion, surgery, anastomosis, fluorescence imaging, parameters

## Abstract

This study tests fluorescence imaging-derived quantitative parameters for perfusion evaluation of the gastric tube during surgery and correlates these parameters with patient outcomes in terms of anastomotic leakage. Poor fundus perfusion is seen as a major factor for the development of anastomotic leakage and strictures. Fluorescence perfusion imaging may reduce the incidence of complications. Parameters for the quantification of the fluorescence signal are still lacking. Quantitative parameters in terms of maximal intensity, mean slope and influx timepoint were tested for significant differences between four perfusion areas of the gastric tube in 22 patients with a repeated ANOVA test. These parameters were compared with patient outcomes. Maximal intensity, mean slope and influx timepoint were significantly different between the base of the gastric tube and the fundus (*p* < 0.0001). Patients who developed anastomotic leakage showed a mean slope of almost 0 in Location 4. The distance of the demarcation of ICG to the fundus was significantly higher in the three patients who developed anastomotic leakage (*p* < 0.0001). This study presents quantitative intra-operative perfusion imaging with fluorescence. Quantification of the fluorescence signal allows for early risk stratification of necrosis.

## 1. Introduction

Patients diagnosed with esophageal malignancies may undergo esophagectomy, removal of the malignant esophagus, and subsequent gastric tube reconstruction: the reconstruction of a functional tube out of stomach tissue. To pull up the constructed ‘gastric-tube’, two of the three most important gastric arteries are ligated. Consequently, perfusion of the gastric tissue solely depends on the right gastroepiploic artery and some branches of the right gastric artery, with a risk of decreased perfusion. Decreased perfusion results in a lack of oxygen, nutrients and an accumulation of metabolic waste, and is considered a major contributing factor for the development of anastomotic leakage postoperatively [1]. Anastomotic leakage unfortunately develops in 5–20% of patients and is associated with high rates of morbidity and mortality (3–4%) [2].

Imaging the fluorescence of an intravenously administered exogenous fluorophore allows visualization of the blood vessels in tissue. Using near-infrared light, the autofluorescence induced by tissue structures is lower than for visible light, thereby facilitating the detection of fluorescent light from deeper tissue layers and blood vessels.

Despite the developments in fluorescence imaging during surgery and previous research on fluorescent imaging techniques [3,4,5], evaluation of the fluorescent signal in quantitative values remains challenging [6,7]. This study focuses on the use of quantitative parameters extracted from the recorded fluorescent signals (F) obtained at four locations during surgery of the gastric tube.

We hypothesize that due to the ligation of arteries and veins, the maximal fluorescent intensity (F_max_) and the mean slope (F_slope_) will be smaller at the fundus compared to the base of the gastric tube. Furthermore, the influx timepoint (τ), the timepoint where the fluorescence intensity starts to increase per location, is expected to be larger at the fundus compared with the base of the gastric tube. We also hypothesize that the distance from the end of the right gastroepiploic artery to the fundus and the distance of the end of the fluorescent signal to the fundus can be indicative for the development of anastomotic leakage [8].

The first objective of this study is to develop and test quantitative perfusion-related parameters obtained by fluorescence imaging during surgery in patients with esophageal cancer, which allow to distinguish four perfusion areas (from biologically normal to reduced perfusion). The second objective is to explore the correlation between the quantitative parameters obtained during surgery and hemodynamic parameters and patient outcome in terms of risk assessment of tissue necrosis and anastomotic leakage, defined by the ECCG classification [9].

## 2. Methods

This study applies to the phase 2A of the IDEAL framework [10]. The STROBE (The Strengthening the Reporting of Observational Studies in Epidemiology) guideline [11] and STARD (Standards for Reporting of Diagnostic Accuracy) statement [12] were used to develop the trial methodology.

### 2.1. Patients

Patients who underwent esophagectomy with gastric tube reconstruction were included in this prospective study between October 2015 and June 2016 (Amsterdam UMC, location Academic Medical Center, Amsterdam, The Netherlands). Surgery was performed by two expert upper-gastrointestinal surgeons (SSG, MIB). Patients were selected for either an Ivor Lewis or McKeown procedure (see Surgical procedure), based upon the location of the esophageal tumor. We included 22 consecutive patients based on our sample size analysis (see Statistical analysis) to create a patient group reflecting reality as well as possible. Approval by the medical ethics committee of the Amsterdam UMC, location Academic Medical Center of Amsterdam and written informed consent was obtained before the start of study inclusion.

### 2.2. Surgical Procedure

In all patients, a two- or three-stage thoracolaparoscopic procedure was performed. Twenty patients underwent an minimally invasive Ivor Lewis procedure (2 stage procedure with an intra-thoracic anastomosis) and two patients underwent a minimally invasive McKeown procedure (3 stage procedure with a cervical anastomosis). Due to the intra-thoracic anastomosis, the gastric tube in an Ivor Lewis procedure is slightly shorter than in a McKeown procedure. Mobilization and vascularization of the gastric conduit is the same in both procedures. In all patients a two-field (abdominal and thoracic) lymphadenectomy was performed. Procedures have been described in detail by Chen, Nguyen and Jones [13,14,15]. In brief, after mobilization of the esophagus and intrathoracic and abdominal lymphadenectomy, ligation of the left gastric artery, part of the right gastric artery, the left epiploic artery and the short gastric vessels was performed. In all patients a 3–4 cm wide gastric tube was reconstructed using a powered ECHOLON FLEX Stapler (Ethicon, Johnson and Johnson Health Care Systems, Bridgewater Township, NJ, USA). The right gastric artery divided just at the base of the gastric tube (3–4 cm from the pylorus). This artery supplies the remains of the lesser curvature, while the right gastroepiploic artery supplies the greater curvature from its origin. The 3–4 cm wide gastric tube was constructed with a linear stapeler outside the patients just before the Fluorescence Imaging. After imaging the gastric tube was pulled-up inside the patient to create an intrathoracic anastomosis using a circular stapler, usually 29 mm, in case of a narrow esophagus 25 mm. After stapling, the anastomosis was oversewn with two sutures and fixated behind a pleural flap and concealed in an omentum-plasty.

Hemodynamic parameters, in terms of mean arterial pressure (MAP), cardiac index (CI), cardiac output (CO), stroke volume (SV) and spO_2_, minute volume (MV), FiO_2_, tidal volume (TV) and respiratory rate (RR) and vasoactive medication at the timing of the fluorescene imaging during surgery, were recorded in a Clinical Report Form (CRF).

### 2.3. Intra-Operative Perfusion Imaging by Fluorescence Imaging

Fluorescence imaging was performed with the Artemis NIR (near-infrared) imaging system (Quest Medical Imaging, Middenmeer, the Netherlands). This system used a ring light with a white light source and a NIR light source at 793 nm to illuminate the tissue. Back reflected light was split, the NIR light was directed to an infrared camera and the visible light to a Bayer color camera. The computer created an overlay of both images. To create vascular contrast, indocyanine green (ICG) was used (ICG Pulsion, 25 mg/10 mL). A disposable drape was placed over the camera before advancing into the sterile operation field. All light in the operation room was switched off to minimize external light reflection.

**Figure 1:**
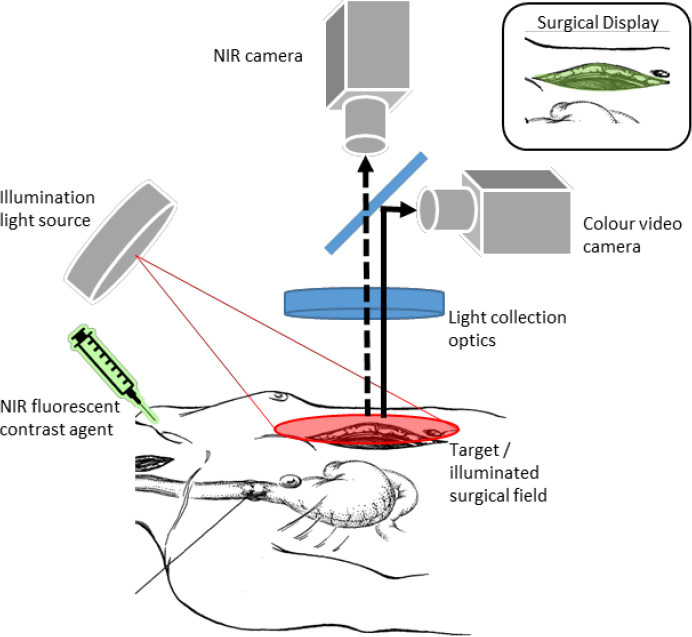
Schematic image of fluorence imaging set-up with two light sources, NIR camera and colour video camera, and ICG injection [16].

Directly after construction, the gastric tube was placed on a sterile surface, still distally attached to the patient, and fluorescence imaging was performed (Figure 1). The Artemis NIR imaging system was placed under an 90 degree angle and at a distance of 40 cm above the gastric tube (measured with the distance laser; Leica Geosystems D110, Germany) and the gastric tube was brought into focus. A metric ruler was placed in the field of view for image calibration and a sterile gauze was placed to point out the watershed area (the end of right gastroepiploic artery). The anesthesiologist administered 2.5 mg ICG intravenously. Perfusion was imaged in continuous video recordings of 2–3 min. No changes were made in location of the anastomosis based on our FI findings, so the FI parameters could objectively be compared with the clinical outcomes.

### 2.4. Data Analysis

**Figure 2:**
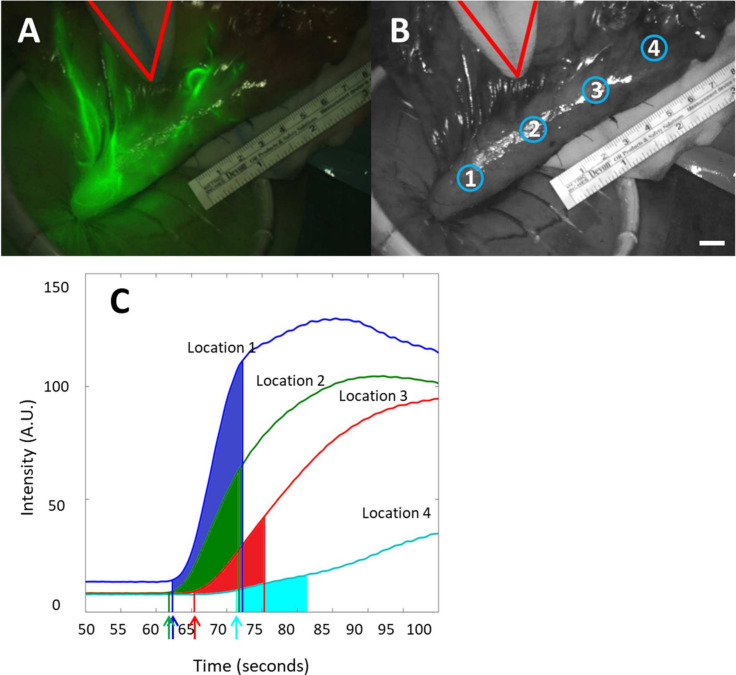
Combined fluorescence and white light image of a gastric tube during surgery with influx of ICG (**A**). In Panel (**B**) a greyscale image of the gastric tube is shown with the four perfusion areas selected in circles with 300 pixels with #1 = 3 cm below the watershed, #2 = watershed, #3 = 3 cm above the watershed and #4 = fundus. In both pictures the sterile gauze (red triangle) is indicating the watershed (end of the right gastroepiploic artery) and the metric ruler is placed in the FOV for pixel calibration (scalebar = 1 mm). Panel (**C**) shows the temporal fluorescence imaging intensity traces for the 4 locations with the colored arrows indicating the automatically detected influx timepoints (arrows) of the curves. Colored areas indicate the 10 s interval.

Software developed at the AMC was used to analyze fluorescence imaging data. Quantitative data analysis was carried out using custom-made scripts written in MATLAB (The Mathworks Inc., Natick, MA, USA). Before measurements in the clinical setting, the illumination profile of the Artermis system was measured in a laboratory setting. Illumination heterogeneity was corrected for in the software. In every patient, four regions of interest (ROIs) of 300 pixels were selected from the base of the gastric tube towards the fundus (from normal to decreased perfusion): location #1 = 3 cm below the watershed, #2 = watershed, #3 = 3 cm above the watershed and #4 = fundus. By calculating the average fluorescent intensity in these ROIs for consecutive images, four region-specific temporal ICG fluorescence intensity profiles (Figure 2) per patient were obtained. Because of the high risk nature of this surgical procedure, the measurement time was maximized to 2–3 min after ICG administration. Because this time was too short to reach a peak in intensity for all locations in all patients, maximal intensity (F_max_) was determined in the range between the influx timepoint and 50 s. In order to quantify the influx of ICG for each location, two parameters were evaluated: (1) The rate at which the fluorescence intensity increased (F_slope_), and (2) the influx timepoint (τ) indicating the time at which the fluorescence intensity was statistically significantly larger than the background. The first parameters are widely used in CT brain perfusion imaging, where the parameter F_slope_ is indicated as ‘cerebral blood flow’ (CBF), the area under the curve is ‘cerebral blood volume’ and the F_max_ is T_max_ [17].

**Figure 3:**
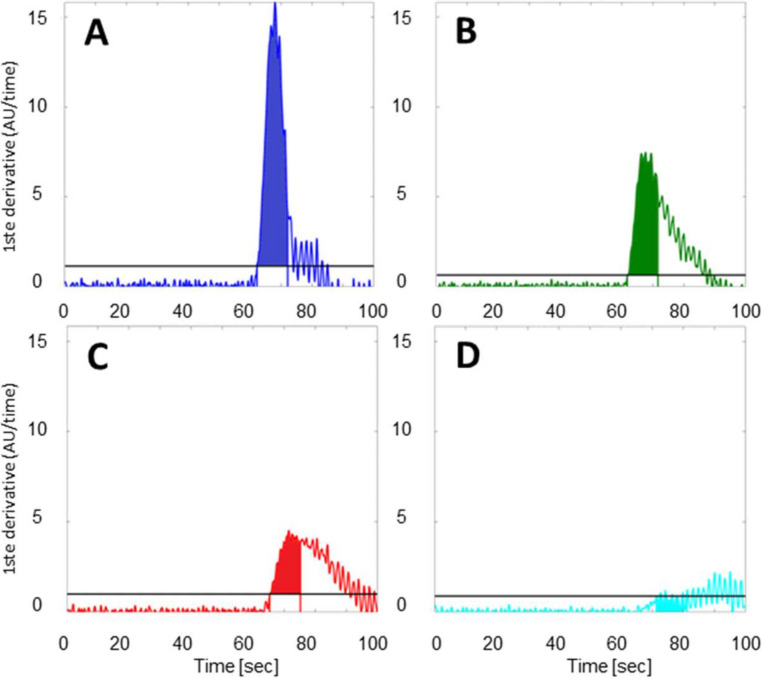
Panel (**A**–**D**) show the point-wise derivative curves of the four intensity traces as a function of time at location #1 (dark blue) (**A**), #2 (green) (**B**), #3 (red) (**C**) and #4 (light blue) (**D**), with colors indicating the corresponding fluorescence trace in Figure 2.

Location-specific mean slopes F_slope_ were computed by performing a succession of signal processing steps. First, a smoothing filter was applied to all temporal fluorescence traces. Here, local regression was employed, using weighted linear least squares and a 1^st^ degree polynomial model with a span of 21 data points assigning zero weight to outliers outside six mean standard deviations. Following this smoothing operation, point-wise derivatives were calculated for all traces (see Figure 3. Next, to enable automatic detection of the location-dependent moment of ICG influx time (τ i.e., the trace-specific inflection points), we calculated the mean (μ_noise,i_) and standard deviation (σ_noise,i_) of the point-wise derivatives in the first 10 s of each of the four derivative curves (i = 1, 2, 3, 4). μ_noise,i_ and σ_noise,i_ then served as trace-specific reference points in a statistical criterion: an initial curve-specific increase in fluorescence intensity was deemed significant if the corresponding point-wise derivative deviated from its respective μ_noise,i_ by more than 5.5 σ_noise,i_ (these significance levels are indicated as horizontal lines in Figure 3). Following this automated influx-detection, four 10 s intervals were defined from the trace-specific influx moments onwards (beginning and ends of which are demarcated by colored vertical lines, in Figure 1, panel C and Figure 3). Using these four sampling intervals, trace-specfic (i.e., location-specific) mean slopes were calculated by averaging the contained point-wise derivatives.

### 2.5. Statistical Analysis

Data were analyzed using Prism (GraphPad Prism Version 5.01, GraphPad Software, San Diego, CA, USA). Study size was based on the detection of hemodynamic change in images, using Hedges’ g. A sample size of 20 patients will have 80% power to detect an effect size of 0.66, using a paired *t*-test with a 0.05 two-sided significance level. Taking 10% missing/unevaluable measurements into account, 22 patients were included. To check data sets for normality a D’Agostino-Pearson test was applied. Quantitative perfusion parameters at four locations were compared by means of a repeated measure ANOVA test. Significance of the differences of these intensity parameters, distances of perfusion demarcation to the fundus and distances of the watershed to the fundus between the leakage group and non-leakage group were measured using a unpaired *t*-test. Patient-specific hemodynamic parameters were tested for correlation with quantitative perfusion-related parameters using a Wilcoxon signed-rank test.

## 3. Results

### 3.1. Participants

In total, 26 consecutive patients who underwent esophagectomy with gastric tube reconstruction signed informed consent and were included in this study between October 2015 and June 2016 (Amsterdam UMC, location Academic Medical Center, Amsterdam, The Netherlands). Four patients were excluded based on the long operation time and therefore did not undergo imaging, and two patients were excluded based on evaluability of images (stability).

Patient characteristics showed the inclusion of 18 men and 2 women with a mean age of 61 and a body mass index of 25.8 kg/m^2^ (Table 1). There was no correlation between hemodynamic parameters (Table 2) and F_max_, F_slope_ or τ.

### 3.2. Feasibility of Fluorescence Imaging

**Figure 4:**
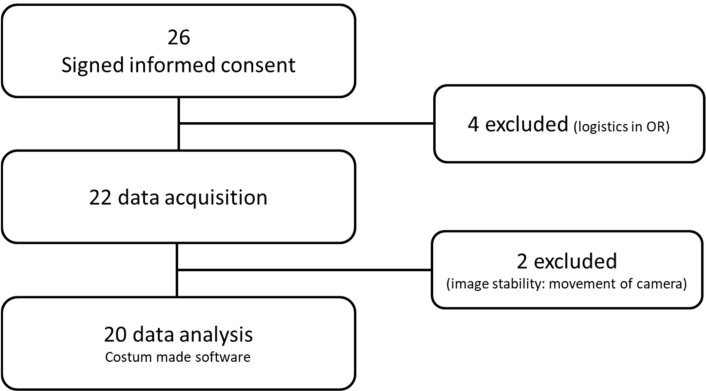
Flow diagram of patient inclusion.

**Figure 5:**
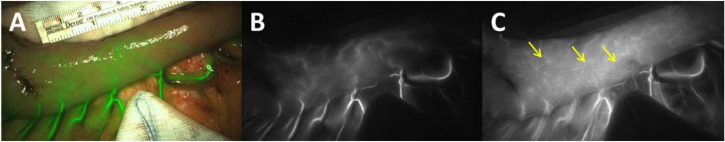
Fluorescence image of the gastric tube during surgery with the overlay image (**A**), the near infrared image at the influx timepoint of ICG (τ), clearly depicting the arteries (**B**). After influx in the arteries, perfusion of the microvascular network (yellow arrows) is visible in the NIR image (**C**) τ + 10 s. The sterile gauze indicates the watershed. Upper scale of the metric ruler is in cm.

Fluorescence imaging of gastric tube perfusion was visible in all patients (*n* = 22). Figure 4 shows a typical NIR fluorescence image and overlay image. In four patients, the fluorescence imaging light intensity settings were adjusted too late, which had to be corrected for during data analysis. In three patients, Location 1 (=3 cm below watershed) was inside the patient and could not be evaluated. In two patients the camera was moved during measurements, and those data therefore had to be excluded (Figure 4). In all analyzed patients (*n* = 20), influx of ICG was visible intra-operatively and the ischemic fundus regions could be successfully differentiated from the non-ischemic perfusion areas (Locations 1 and 2) since the influx of ICG was later and less intense. Additionally, at the end of the measurement the accumulation of ICG was still visible in the fundus area due to the decrease in clearance by the ligated veins. Fluorescence images were easy to obtain because of the non-contact set-up in the OR. Obtained images depicted small blood vessels after the influx of ICG by the arteries (Figure 5).

### 3.3. Quantitative Perfusion Imaging with FI

**Figure 6:**
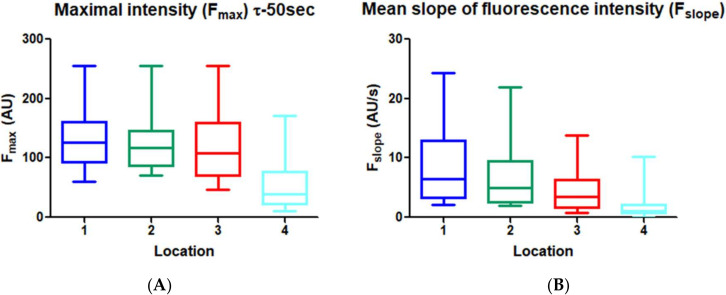

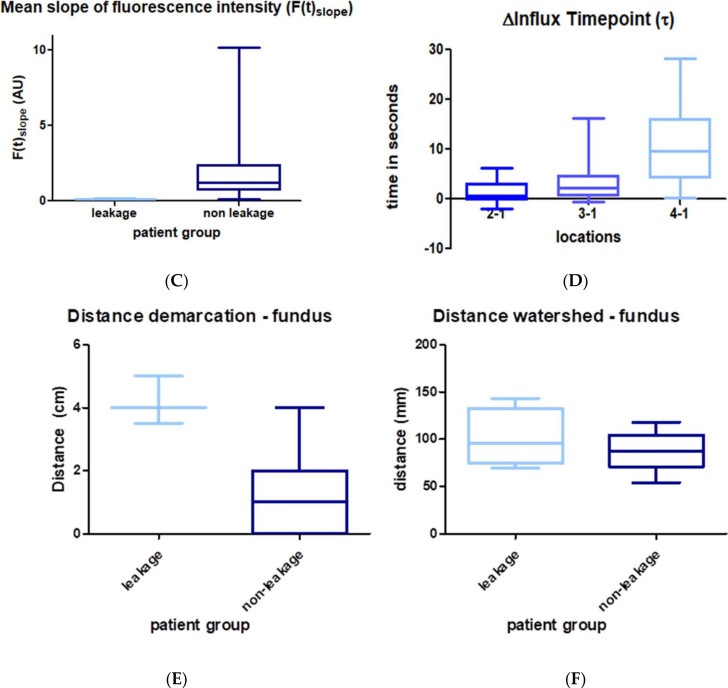
Panel (**A**): Maximal intensity F_max_ of ICG and Panel (**B**): mean slope of fluorescence intensity over time (F_slope_) measured with the custom-made software at the four perfusion areas. Panel (**C**): Mean slope of fluorescence intensity at Location 4 for patients who developed leakage (3) versus patients without leakage (17). F_slope_ was <0.2 in patients who developed leakage. Panel (**D**): Influx timepoint (τ) in seconds of all patients with Location 1 imaged (*n* = 16). Panel (E): Measured distance between demarcation of ICG and fundus in cm, differences are shown between the patients with leakage (*n* = 3) and the non-leakage group (*n* = 17). The distance was smaller in the non-leakage group (*p* = 0.0005). Panel (**F**): Measured distance of the watershed area (selected by the surgeon based on arterial pulse and visualization, pointed out with a sterile gauze) in cm. No significant differences were present between the leakage group and non-leakage group (*p* = 0.30). Data in all patients are presented in boxplots with median, interquartile ranges and maximum and minimum values.

In Figure 6A the results of F_max_ of ICG measured with the custom-made software are shown in boxplots with median, interquartile ranges, and maximum and minimum values. F_max_ was significantly different at Location 4 (*p* < 0.0001) compared with Locations 1, 2 and 3, with a lower intensity value at Location 4 (51 ± 39) in contrast to Locations 1, 2 and 3 (129 ± 49, 129 ± 56, 123 ± 64). The F_max_ were not significantly different between Locations 1, 2 and 3.

The mean slope of the fluorescent intensity over time F_slope_, as depicted in Figure 6B, was significantly lower at the fundus (Location 4) compared with Locations 1, 2 and 3 (*p* = 0.0002, *p* = 0.0003 and *p* < 0.0001). Moreover, in patients 1, 6 and 9, the mean slope was almost zero at Location 4. Interestingly, from these patients, patients 1 and 6 developed anastomotic leakage (Figure 6C). In patient 9, Location 4 was measured exactly on the stapler side, and therefore intensity was low. The gastric tubes of patients 5, 18 and 20 were shorter in length, and therefore, Location 1 was located intra-thoracically and could not be imaged.

The third quantitative parameter τ, the time point where the fluorescence intensity started to increase per perfusion area, was higher at Location 4 compared with Locations 1, 2 and 3 (*p* < 0.0001, *p* < 0.0001 and *p* = 0.0009) (Figure 6D).

In eight patients, fluorescence was clearly visible in the whole gastric tube, while in 14 patients, a clear demarcation of fluorescence intensity at the end of the recording time was visible. The distance of this demarcation to the fundus, measured with the custom-made software, was larger for patients who developed anastomotic leakage (*p* = 0.0005, *n* = 3), (Figure 6E). The watershed area, at the end of the right gastroepiploic artery, was selected by the surgeon based on arterial pulse pressure and visualization. The distance of the watershed area to the fundus did not differ between the leakage and non-leakage groups, and therefore does not seem to be a predictive parameter for the occurance of anastomotic leakage (Figure 6F).

## 4. Discussion

This study demonstrates the feasibility of fluorescence imaging to evaluate perfusion in terms of quantitative perfusion-related parameters. A significant difference was observed in terms of maximal intensity F_max_, mean slope F_slope_ and influx timepoint τ between the fundus (Location 4) and the other three perfusion areas. Moreover, F_slope_ < 0.2 and an increased distance of the demarcation towards the fundus tip were found in patients who developed anastomotic leakage (*n* = 3). These findings demonstrate potential parameters in quantification of the fluorescence imaging signal that could help the surgeon in perfusion evaluation for the prediction of anastomotic leakage development. The observed lack of difference in the distance of the watershed area to the fundus between the leakage and non-leakage groups, means that this distance does not seem to be a predictive parameter for the occurance of anastomotic leakage.

Multiple risk factors have been studied for the development of anastomotic leakage, such as age, nutritional state and radiation. Additionally, the duration of the operation, blood transfusion, tumor stage and location and type of reconstructions have a significant impact on post-operative recovery, morbidity and mortality. Perfusion is still seen as the major contributing factor in the development of anastomotic leakage and success in anastomosis dependson a good perfusion state. Fluorescence imaging has been studied increasingly in the past decade, especially in cancer surgery for the intra-operative imaging of malignant tissue and sentinel nodes [18]. However, a lack of data interpretation by missing quantitative perfusion-related parameters inhibits the implementation of fluorescence imaging for standard clinical practice.

In esophageal surgery, previous research focused on the qualitative imaging of perfusion in terms of ‘good vs. sparse or absent’ blood flow and detection of blood flow routes [3,4,5,6]. Yukaya et al. measured perfusion quantitatively as luminance over time, however they did not find any significant correlation with decreased perfusion and anastomotic leakage [7]. Yamaguchi et al. used fluorescence time to decide the location of anastomoisis and found good results in an Randomized Control Trial (RCT) with a low number of anastomic leakages [19] Zehetner et al. measured the distance of demarcation assessed by fluorescence imaging towards the anastomosis and also found a significant correlation with leakage [8]. In the area after the demarcation, there is no perfusion, no viability of cells and necrosis occurs [20]. Jafari et al. retrospectively showed the decrease in leakage complications after use of fluorescence imaging [21]. Additionally, the tension on the anastomotic site is also an important factor, as described by Kitagawa et al. [22]. The fluorescence quantitative parameter could be good (at location one or two for example), but if the gastric tube is too short, tension on the anastomosis will be so high that ulceration will develop. Surgeons should take this into account.

Fluorescence imaging gives a real-time wide field image and therefore the evaluation of the fundus can be compared with the base of the gastric tube in the same image. The Artemis system in this study gives an overlay of the NIR image and the video image, enabling real-time perfusion evaluation. The evaluation makes the microcirculation visible, however the image interpretation during surgery is still subjective. Custom-made software enabled us to objectively look at the fluorescence images of gastric tube perfusion in terms of maximal intensity, mean intensity slope and influx time. Comparable parameters are widely used in software for CT perfusion imaging (T_max_, Cerebral Blood Flow (CBF) and Mean Transit Time (MTT)), which points out the valuable implementation of these perfusion parameters in the clinic. The study in this manuscript used offline analysis methods to find and optimize quantitative fluorescence parameters. After the study presented here, a follow-up study was executed (clinical trials NL8527) to test these parameters in a larger patient group, while using intra-operative real-time software. With these parameters, we hopefully show how FI can be used to (A) find the best anastomic side and (B) show that these parameters influence the patient outcome.

Objective fluorescence interpretation by quantitative parameters can help the surgeon: (1) in the correct placement of anastomosis in well perfused tissue, (2) to decide to administer fluid or medication for perfusion improvement, (3) to improve perfusion by bloodletting of the short gastric veins or by creating an extra arterial vessel anastomosis, (4) to select high risk patients with comorbidities and a decreased perfusion in the anastomotic area for strict post-operative monitoring by barium swallowing and endoscopy [23,24].

Because the fluorescence images are made without tissue contact, this technique can easily be used in the clinic during operations, without intervening in the sterile field. However, still some limitations exist. First, lights needed to be be switched off in the OR to decrease back reflection, which is a disadvantage for surgeons in short time operation settings, especially for such a critical operation with a high risk of complications. The imaging time consequently was limited to 2–3 min. Off-line data analysis revealed that this imaging time was not sufficient to reach the maximal intensity in all locations, which can be attributed to impaired perfusion and ligations of arteries in the fundus area. The maximal intensity between the influx timepoint plus 50 s still gave us the opportunity to evaluate the fluorescence imaging signal quantitatively. Additionally, due to ligation of veins, venous engorgement developed, as is described before by Jansen et al. [25]. The engorgement of veins is thought to result from the impaired outflow of blood that will lead to accumulation of ICG in the fundus area, which wil influence the maximal intensity.

Secondly, the illumination of the tissue was not homogeneous, inducing low fluorescence intensity values in the corners of the image. This inhomogeneous illumination profile can be corrected for in the analysis of the signals, as we did in the laboratory and built-in in our custom-made software, but was visible during surgery. These lower values may result in a false observation of less perfusion in the corners of the image, an artefact that surgeons should be aware of. In the future, flat panel tissue illumination could solve this problem. Additionally, one patient showed very low fluorescence intensity values at the fundus, without the development of anastomotic leakage. In this patient the intensity was measured on top of the stapler line, which resulted in low perfusion-related parameters. Clearly, these artefacts should be avoided or ignored during surgery.

Thirdly, in this study, only a small number (*n* = 3) of patients developed anastomotic leakage, which hampers our ability to draw final conclusions. We did observe extremely low measurements of F_slope_ at the fundus in the leakage patients (F_slope_ < 0.2). In contrast to other studies, the surgical design was not changed by the interpretation of fluorescence imaging, since it was desired to objectify the correlation of the fluorescence imaging-derived perfusion-related parameters and anastomotic leakage.

## 5. Conclusions

We presented quantitative perfusion imaging based on intravenously administered ICG during esophagectomy with gastric tube reconstruction. Influx of perfusion with fluorescence imaging was visible in all patients. The quantitative perfusion-related parameters of maximal intensity, mean intensity slope, and influx timepoint were significantly lower at the fundus. A low slope and a longer distance of the dermarcation of fluorescence intensity towards the fundus were found in patients with anastomotic leakage, potentially allowing early objective evaluation of impaired gastric microcirculation. These parameters could help the surgeon in choosing the optimal anastomotic site and preventing necrosis, which will reduce post-operative complications.

## Figures and Tables

**Table 1:** Patient characteristics. COPD = chronic obstructive pulmonary disease.

Patient Characteristics	
Age—yr	
median	62
range	37–79
Gender—m, f (n)	19, 1
Body mass index, kg/m^2^	
Median	25.9
Range	17–34.2
Procedures—no.(%)	
Ivor Lewis	19 (95)
McKeown	1 (5)
Duration of operation—mean, sd (min)	449 (46)
Blood transfusion—no. (%)	1 (5)
Tumor stage—no. (%)	
T1	0 (0)
T2	4 (20)
T3	15 (75)
Tumor location—no. (%)	
Mid esophagus	2 (10)
Distal esophagus	18 (90)
Cardiovascular disease—no. (%)	7 (35)
Diabetes Mellitis 1—no. (%)	0 (0)
Diabetes Mellitis 2—no. (%)	2 (10)
COPD—no. (%)	2 (10)

**Table 2:** Parameters (mean arterial pressure (MAP), heart rate (HR), systolic blood pressure (SBP), diastolic blood pressure (DBP), cardiac output (CO), stroke volume (SV), stroke volume variation (SVV), and cardiac index (CI).

Hemodynamic Parameters (Mean, SD)	
MAP (mm Hg)	71 ± 9
HR (beats/min)	85 ± 14
SBP (mm Hg)	106 ± 17
DBP (mm Hg)	65 ± 16
Temperature nasopharyx (°C)	36.2 ± 0.6
CO (L/min)	6.9 ± 2.3
SV (mL)	75.4 ± 18.5
SVV (%)	7.8 ± 2.6
CI (L/min m^2^)	3.1 ± 0.9

## Data Availability

Data was archived at the Department of Biomedical Engineering and Physcis, Amsterdam UMC, location AMC, L0, Amsterdam, The Netherlands.
